# Genetic association between sow longevity and social genetic effects on growth in pigs

**DOI:** 10.5713/ajas.18.0789

**Published:** 2019-02-07

**Authors:** Joon Ki Hong, Yong Min Kim, Kyu Ho Cho, Eun Seok Cho, Deuk Hwan Lee, Tae Jeong Choi

**Affiliations:** 1 National Institute of Animal Science, Rural Development Administration, Cheonan 31000, Korea; 2 Department of Animal Life Resources, Hankyong University, Anseong 17579, Korea

**Keywords:** Indirect Genetic Effects, Longevity, Pigs, Stayability, Social Genetic Effects

## Abstract

**Objective:**

Sow longevity is important for efficient and profitable pig farming. Recently, there has been an increasing interest in social genetic effect (SGE) of pigs on stress-tolerance and behavior. The present study aimed to estimate genetic correlations among average daily gain (ADG), stayability (STAY), and number of piglets born alive at the first parity (NBA1) in Korean Yorkshire pigs, using a model including SGE.

**Methods:**

The phenotypic records of ADG and reproductive traits of 33,120 and 11,654 pigs, respectively, were evaluated. The variances and (co) variances of the studied traits were estimated by a multi-trait animal model applying the Bayesian with linear-threshold models using Gibbs sampling.

**Results:**

The direct and SGEs on ADG had a significantly negative (−0.30) and neutral (0.04) genetic relationship with STAY, respectively. In addition, the genetic correlation between the social effects on ADG and NBA1 tended to be positive (0.27), unlike the direct effects (−0.04). The genetic correlation of the total effect on ADG with that of STAY was negative (−0.23) but non-significant, owing to the social effect.

**Conclusion:**

These results suggested that total genetic effect on growth in the SGE model might reduce the negative effect on sow longevity because of the growth potential of pigs. We recommend including social effects as selection criteria in breeding programs to obtain satisfactory genetic changes in both growth and longevity.

## INTRODUCTION

Sow longevity is most important factor for production efficiency and profitable pig farming. To ensure profitability for the producer, a sow should produce at least three litters before culled. The optimal time to replace a sow depends on factors such as the sow’s performance, its housing, feeding, and insemination costs, and the cost and genetic merit of the replacement gilt compared to the sow being replaced [1]. Early culling results in less piglets born alive over the sow’s lifetime and leads to irregular replacement of sows. The longevity can be measured using stayability (STAY) to a certain age. STAY is the ability of a sow to survive until a specific parity in its life and is related to the number of piglets born alive over the lifetime [2]. Several studies estimated the heritability of STAY in sows and tried to find its early indicators, such as leg conformation [2–5]. Sows can be stressed in various stages (e.g., mating, gestation, and farrowing). Maternal stress during gestation can affect the physiological development of suckling piglets [6]. Low reproductive performance due to these stresses can result in the early culling of sows.

Recently, there has been an increasing interest in the relationship of social interaction of pigs with stress-tolerance and behavior. The genetic effect of an individual on the phenotypes of its social partners (i.e., pen mates) is often termed social genetic effect (SGE) or the indirect genetic effect [7]. The report by Bergsma et al [8] on pigs indicated that the heritable social interaction among various group members might play a role in their average daily gain (ADG). Canario et al [9] improved this SGE model by accounting early-life environmental effects to avoid bias in the estimated genetic parameters for social effects. In addition, the importance of SGE can be recognized from many previous reports [10–12], which showed that the higher social breeding value and some desirable characteristics in pigs, i.e., fearlessness and stress-tolerance, are associated with each other.

In the present study, we aimed to include the social effects in a model for estimating the genetic correlations of ADG and STAY with the number of piglet born alive at the first parity (NBA1) in Korean Yorkshire pigs.

## MATERIALS AND METHODS

### Ethics statement

In this study, Animal Care and Use Committee approval was not necessary because data were obtained from weight and reproductive records in an existing database.

### Animal phenotypes

Data were provided by Sunjin, the Korean company for pig genetic evaluation (Yorkshire Sunjin, Danyang, Korea, http://dad.fao.org/). The breeder had selected pigs focused on reproductive traits, such as litter size. Pedigree was traced back 3 generations and included 37,858 animals with 385 sires, and 2,520 dams. A total of 37,745 animals with both sire and dam known were included.

The phenotypic dataset on the growth rate of animals was obtained from performance tests of Yorkshire pigs born between 2001 and 2015 (*N* = 33,120). A total of 4 to 14 pigs of the same gender were kept in each pen to form the groups of pigs and the average group size was 8.2±2.0 pigs. The space allowance per pig was 0.8 to 1.2 m^2^ at the start of the performance test. The pigs were fed *ad libitum* and water was constantly accessible through nipple drinkers. The feeding program was applied in accordance with pig testing standards of the Korean Animal Improvement Association (http://www.aiak.or.kr/eng/index.jsp). The performance evaluations of the ADG of pigs started soon after each animal reached a live body weight of 30 kg and continued until a target weight of 90 kg was attained. On average, fewer than 160 days was required to attain this target weight. The average ADG was recorded to be 802±93 g/d.

The phenotypic records of the reproductive traits for 11,654 pigs born between 2001 and 2014 were evaluated in this study. Gilts at selection were contemporaries and managed under the same conditions (a single pen). Age at first mating of gilts was typically 230 to 250 d and mating was conducted twice (24 h and 36 h after mounting) by artificial insemination. All the naturally farrowed sows had a lactation period of 24 to 28 days. The breeder culled the pigs that showed repeated gestation failure or low reproductive performance. The average culling parity was 4.96±2.85, with a range of 1 to 15. STAY, binary response variable defined as the ability of a sow to survive until its second parity. The average STAY was recorded to be 1.85±0.35. NBA1 was recorded to be 10.38±2.84 piglets.

### Statistical analysis

The variances and (co) variances of the studied traits were estimated by an animal multi-trait model applying the Bayesian with linear-threshold models using Gibbs sampling. For ADG, the effects of birth year-month (168 levels), sex (male or female), and group size (11 levels) were fitted as fixed effects. In the model, age and age squared at target weight were fitted as covariates. The models also included the random effects of group identity (4,927 levels) and birth litter (8,712 levels). We accounted social early-life environmental effects (birth litter of piglets within a group) in the model to avoid bias in the estimated genetic parameters for social effects [9]. For STAY and NBA1, the effects of farrowing year-month at first parity (169 levels) were used as fixed effects and birth litter (5,365 levels) was used as a random effect.

Animals were fitted as a random effect in the model. The statistical model for each group of traits is presented below:

$$\begin{array}{l} \text{y}=\text{Xb}+{\text{Z}}_{\text{D}}{\text{a}}_{\text{D}}+{\text{Z}}_{\text{S}}\;{\text{a}}_{\text{S}}+{\text{W}}_{\text{c}}+\text{Vg}+{\text{T}}_{\text{pe}}+\text{Ul}+\text{Qk}+\text{e}(\text{ADG}),\\ \text{y}=\text{Xb}+{\text{Z}}_{\text{D}}{\text{a}}_{\text{D}}+\text{Ul}+\text{e}(\text{STAY},\text{NBA}1), \end{array}$$

where, y is the vector of observations, b is the vector of fixed effects, a_D_ is the vector of direct genetic effects (DGE), a_S_ is the vector of SGE, g is the vector of random group, where $g\sim N(0,I\sigma_{g}^{2})$, l and k are the vectors of random birth litter and social early-life environment effect, respectively, and e is the vector of residuals, where, $e\sim N(0,I\sigma_{e}^{2})$. X, Z_D_, Z_S_, W, V, T, U, and Q are the corresponding incidence matrices. To account for the differences in group size, as suggested by Canario et al [9], an additional covariate term known as dilution $\left(\frac{\text{Average group size}-1}{\text{Group size}-1}\right)$ was added to the SGE and early-life environmental effects. DGE and SGE had the following multivariate normal (MVN) distribution:

$$\left[\begin{array}{c} a_{D(ADG)}\\ a_{S(ADG)}\\ a_{D(STAY)}\\ a_{D(NBA1)} \end{array}\right]\sim \text{MVN }(0,C⊗A),$$

in which C is defined by the matrix,

$$\left[\begin{array}{cccc} \sigma_{a_{D(ADG)}}^{2} & \sigma_{a_{D(ADG)}a_{S(ADG)}} & \sigma_{a_{D(ADG)}a_{D(STAY)}} & \sigma_{a_{D(ADG)}a_{D(NBA1)}}\\ \sigma_{a_{D(ADG)}a_{S(ADG)}} & \sigma_{a_{S(ADG)}}^{2} & \sigma_{a_{D(STAY)}a_{S(ADG)}} & \sigma_{a_{D(NBA1)}a_{S(ADG)}}\\ \sigma_{a_{D(ADG)}a_{D(STAY)}} & \sigma_{a_{D(STAY)}a_{S(ADG)}} & \sigma_{a_{D(STAY)}}^{2} & \sigma_{a_{D(STAY)}a_{D(NBA1)}}\\ \sigma_{a_{D(ADG)}a_{D(NBA1)}} & \sigma_{a_{D(NBA1)}a_{S(ADG)}} & \sigma_{a_{D(STAY)}a_{D(NBA1)}} & \sigma_{a_{D(NBA1)}}^{2} \end{array}\right],$$

where, $\sigma_{a_{D}}^{2}$ is variance of direct breeding values (DBV) for each trait, $\sigma_{a_{S}}^{2}$ is variance of social breeding values (SBV) for ADG, *σ_a_*_*_D_*_*_a_*_*_S_*_ is the covariance between DBV and SBV on same or different traits, *σ_a_*_*_D_*_*_a_*_*_D_*_ is the covariance between different DBV, A is pedigree-based relationship matrix, and C⊗A denotes the Kronecker product of two matrices.

The birth litter effect and social early-life environment effect had the following MVN distribution:

$$\left[\begin{array}{c} l_{\left(ADG\right)}\\ k_{\left(ADG\right)}\\ l_{\left(STAY\right)}\\ l_{\left(NBA1\right)} \end{array}\right]\sim \text{MVN }(0,K⊗I),$$

in which K is defined by the matrix,

$$\left[\begin{array}{cccc} \sigma_{l_{\left(ADG\right)}}^{2} & \sigma_{l_{\left(ADG\right)}k_{\left(ADG\right)}} & \sigma_{l_{\left(ADG\right)}l_{\left(STAY\right)}} & \sigma_{l_{\left(ADG\right)}l_{\left(NBA1\right)}}\\ \sigma_{l_{\left(ADG\right)}k_{\left(ADG\right)}} & \sigma_{k_{\left(ADG\right)}}^{2} & \sigma_{l_{\left(STAY\right)}k_{\left(ADG\right)}} & \sigma_{l_{\left(NBA1\right)}k_{\left(ADG\right)}}\\ \sigma_{l_{\left(ADG\right)}l_{\left(STAY\right)}} & \sigma_{l_{\left(STAY\right)}k_{\left(ADG\right)}} & \sigma_{l_{\left(STAY\right)}}^{2} & \sigma_{l_{\left(STAY\right)}l_{\left(NBA1\right)}}\\ \sigma_{l_{\left(ADG\right)}l_{\left(NBA1\right)}} & \sigma_{l_{\left(NBA1\right)}k_{\left(ADG\right)}} & \sigma_{l_{\left(STAY\right)}l_{\left(NBA1\right)}} & \sigma_{l_{\left(NBA1\right)}}^{2} \end{array}\right],$$

where, $\sigma_{l}^{2}$ is variance of birth litter for each trait, $\sigma_{k}^{2}$ is variance of social early-life environment effect for ADG, $\sigma_{lk}^{2}$ is the covariance between birth litter and social early-life environmental effect on the same or different traits, $\sigma_{ll}^{2}$ is the covariance between birth litter on different traits, and I is an identity matrix of appropriate dimensions.

According to Bijma [7], for traits affected by heritable social effects, the variance of total breeding values (TBV) for ADG represents the total heritable variation that is exploitable for selection. The TBV of the *i* animal is defined as follows:

$${TBV}_{i}=a_{D,i}+(n-1)a_{S,i,}$$

where, *n* indicates the average size (8.2 pigs) of social groups. The TBV is the heritable effect of an individual on trait values in the population, which is the sum of the individual’s DBV (*a_D_*_,_*_i_*) of its own phenotype and the SBV (*a_S_*_,_*_i_*) of the phenotypes of its *n*–1 group mates. Moreover, Bijma [7] stated that the total heritable variance ($\sigma_{TBV}^{2}$) determines the population’s potential response to selection and can be expressed as:

$$\sigma_{TBV}^{2}=\sigma_{aD}^{2}+2(n-1)\sigma_{aDaS}+{\left(n-1\right)}^{2}\sigma_{aS}^{2}.$$

According to Canario et al [9], the phenotypic variance ($\sigma_{P}^{2}$) for ADG for such a model can be calculated as follows:

$$\sigma_{p}^{2}=\sigma_{aD}^{2}+(n-1)\sigma_{aS}^{2}+\sigma_{g}^{2}+\sigma_{l}^{2}+(n-1)\sigma_{k}^{2}+\sigma_{e}^{2}.$$

The total heritable variance for ADG can be expressed relative to phenotypic variance [13] as follows:

$$T^{2}=\sigma_{TBV}^{2}/\sigma_{P}^{2}.$$

For STAY and NBA1, heritability estimates were calculated according to the equations below:

$$h^{2}=\sigma_{a_{D}}^{2}/(\sigma_{a_{D}}^{2}+\sigma_{l}^{2}+\sigma_{e}^{2}).$$

The covariance of between TBV for ADG (*TBV*_ADG_) and DBV of STAY or NBA1 (*DBV*_REP_) was computed as:

$$\begin{array}{l} Cov\;({TBV}_{\text{ADG}},{DBV}_{\text{REP}})\\ =\sigma_{a_{D(ADG)}a_{D(REP)}}+(n-1)\sigma_{a_{D(REP)}a_{S(ADG)}}. \end{array}$$

To fit a model, THRGIBBS1F90 with Bayesian inference using Gibbs sampling was used [14]. The Gibbs samplers were run as single chains of 1,200,000 rounds. The first 600,000 rounds discarded as burn-in thinning every 100 samples. This resulted in 6,000 samples being used for post-Gibbs analyses completed using POSTGIBBSF90 [14]. Significant difference from zero was based on the highest posterior density, which signifies a 95% confidence interval for the estimate (p<0.05).

## RESULTS AND DISCUSSION

### Heritability estimation

The (co)variances and parameters obtained from the model studied are presented in Table 1. All genetic variances were significantly larger than zero.

*Average daily gain in social genetic effect model*: The *T*^2^ estimates for ADG was 0.48±0.05 and were greater than classical heritability (*h*^2^ = 0.34±0.02). The contribution of social genetic variance ($7.2\times \sigma_{a_{S}}^{2}$) was 27% of the total heritable variance ($\sigma_{TBV}^{2}$). Notably, even when the social variance was markedly smaller than the direct genetic variance, its contribution to $\sigma_{TBV}^{2}$ was largely due to the factor (n–1)^2^. Our *T*^2^ estimates with high *h*^2^ for ADG was greater than those of prior studies [9,13] and coincided with those of Duijvesteijn [15]. In this study, the breeder focused on only reproductive traits for pig selection, such as litter size, which might be affected by the substantial genetic variation for growth n the population.

Social early-life environmental variance ($\sigma_{k}^{2}=32\pm 5$) was greater than social genetic variance ($\sigma_{a_{S}}^{2}=18\pm 5$). This result was consistent with those of Canario et al [9] in that individuals experienced early in life affected their social skill in adulthood. Sociable pigs in early-life could solve dominance conflicts with unfamiliar pigs in adult life more quickly when they occurred [16]. Therefore, social effects are due to both genetic and social early-life environmental effects.

#### Stayability

The estimated heritability for STAY of the present study was 0.07±0.02. The heritability in the present study for STAY agreed with low to moderate heritability (0.06 to 0.18) reported in other studies [2,5,17,18]. Heritability of STAY in the threshold model normally showed greater heritability values than a normal linear model [2,5,18]. A threshold model was used as another form of analysis but the estimated heritability for STAY of the present study was slightly lower than previous results [5,18,19]. Particularly, the birth litter variance (0.19±0.04) was greater than the direct genetic variance (0.10 ±0.03) in STAY and equaled 15% of the phenotypic variance in STAY. In the present study, the rate of culled pigs before the second parity (STAY = 1) was lower (15%) than those of the other studies [2,5,17–19]. Therefore, the low frequency of STAY value 1 may result in slightly lower heritability.

#### Number of piglets born alive at the first parity

The estimated heritability for NBA1 of the current study was 0.10±0.01 (Table 2). This result was in agreement with those of Engblom et al [2]. In addition, they coincided with those for the total number of piglets born in the first litter of other studies [1,20].

### Genetic correlations

The estimated genetic correlations from the multivariate analysis are presented in Table 3.

#### Direct breeding value and social breeding values of average daily gain

The genetic correlation coefficient between DBV and SBV of ADG was neutral (0.03±0.11). This result strongly agreed with the study of Bergsma et al [8], in that the absence of conflicts between an individual’s own growth and mate’s growth might be a consequence of neutral or slightly cooperative social interactions. Moreover, Canario et al [9] suggested that a social effect on the growth rate of a group mate had no cost for the individual studied. In addition, the positive or neutral relationship between direct and SGE is likely to increase the total heritable variation [7,8].

#### Stayability and number of piglets born alive at the first parity

The genetic correlation coefficient between STAY and NBA1 was significantly positive (0.31±0.16), indicating that the breeder in the current study culled sows based on NBA1. Low reproductive performance is a major reason for culling of sows [19,21]. The risk of culling is greater between the first and second litters than between the second and third litters [17]. Therefore, piglet production, particularly at first parity, is an important factor affecting the breeder’s decision to cull sows after first farrowing. In addition, Engblom et al [2] reported that the estimated breeding values for NBA1 had a moderate correlation (0.25) with the number of piglets born alive over a lifetime, after accounting for censoring.

#### Direct breeding value of average daily gain, stayability, and number of piglets born alive at the first parity

The estimated genetic correlation coefficient between DBV of ADG and STAY was significantly negative (−0.30±0.13), suggesting that animals with high genetic potential for ADG could have a lower STAY. However, the genetic correlation coefficients between the DBV of ADG and NBA1 were neutral (−0.04± 0.07). Although genetic relationship between individual growth and the NBA1 was weak, the pigs with high growth can be at risk of early culling, genetically. Therefore, this risk may be more because of factors other than low piglet production at first parity. The high individual growth rate can cause leg problems such as osteochondrosis [22,23]. In this connection, leg problem is a main factor for culling sows and several studies suggest that leg conformation traits in a growth stage could be good early indicators of STAY [3–5,24–26].

#### Social breeding value of average daily gain, stayability, and number of piglets born alive at the first parity

The genetic correlation between the SBV of ADG and STAY was neutral (0.04 ±0.32), whereas that between the SBV of ADG and NBA1 tended to be positive (0.27±0.17), which ranged from −0.05 to 0.60. Maternal stress during gestation can affect the physiological development of suckling piglets [6]. Previous studies showed that higher SBV and some desirable characteristics in pigs, i.e., fearlessness and stress-tolerance, were associated with each other [11,12,27]. Higher SBV could occur because of the apathy of the animal, resulting in reduced negative effects on the growth of others [10,16]. Therefore, the pigs with this apathy may be less affected by various environments. Bailey et al [20] suggested that selection due to SGEs might push distinctive evolutionary dynamics in behaviors, such as competition, cooperation, or reproductive interactions.

#### Total breeding value of average daily gain, stayability, and number of piglets born alive at the first parity

The genetic correlation between TBV of ADG and STAY was negative (−0.23 ±0.20), but non-significant. Owing to the effect of SBV, this was weaker than the genetic correlation between DBV of ADG and STAY (−0.30±0.13). In addition, the genetic correlation between TBV of ADG and NBA1 (0.11±0.11) was more positive than that between DBV of ADG and NBA1 (−0.04±0.07). The associative component of TBV is expressed not only in individual growth, but also in the growth of group mates [7]. Notably, because each pig interacts with its group mates, TBV of a pig is the sum of DBV and the number of group mates× SBV [8]. In Table 2, the contribution of DBV variance (68%) to TBV was greater than that of SBV variance (27%). Although DBV had more effects than SBV on the genetic correlations of the TBV of ADG with that of other traits, SBV can potentially reduce a negative effect on the DBV of STAY. It was concluded that selection for SGEs on growth could be combined with selection not only for individual growth traits but also for longevity traits of pigs in the absence of antagonistic genetic correlations.

### Litter environmental correlation

The correlations of birth litter and social early-life environmental effects are presented in Table 4. Overall, they were relatively low and mostly non-significant. Significant positive correlations were found between birth litter and social early-life environmental effect on ADG (0.43±0.11), which differed from the neutral genetic correlation between DBV and SBV for ADG (0.03±0.11; Table 3). This result indicated that social effects from early-life experiences were dependent on birth litter environment. In other words, positive birth litter environment for growth rate increases social skills later in life. The correlation between birth litter and social early-life environmental effects on ADG was much higher than that of Canario et al [9]. However, our result also suggested that pigs with high social effects on the growth of a group mate did not incur a cost for itself.

A significant negative correlation was found between birth litter effects on ADG and STAY (D_ADG_ – D_STAY_ = −0.38±0.13; Table 4), suggesting that pigs in positive litter environment for its growth rate had a poorer STAY. This result was similar to the negative genetic correlation between DBV of ADG and STAY (DBV_growth_ – DBV_STAY_ = −0.30±0.13; Table 3). Canario et al [9] demonstrated that the social skills that pigs develop in their litter environment have a long-lasting effect on the growth of social partners. In addition, previous studies on STAY accounted for the effect of litter on the estimating paraeter [2,17,19]. The early-litter environment can affect phenotype later in life in terms of both growth and longevity traits, and there can be positive or negative correlation between these traits in the litter environment. Therefore, accounting for multivariate litter effect is required in multi-trait estimation of growth rate and STAY.

## CONCLUSION

When the heritability of ADG was considered as a SGE, it showed 14% higher value than in a normal model. This can lead to a high genetic gain in swine breeding program. The direct effect on growth rate had a negative genetic relationship with the STAY of sows but social effect on growth rate had a neutral genetic relationship. In addition, the genetic relationship between social effects on ADG and NBA1 tended to be positive, unlike the neutral correlation of the direct effects on ADG and NBA1. Therefore, accounting for social effect is essential for the estimation of growth rate, and selection of the TBV of growth rate might reduce the negative effects of STAY on the individual growth potential of pigs. In addition, there can be positive or negative correlation between direct effect and social effect on growth rate and STAY in the litter environment, suggesting that accounting for multivariate litter effects is important for multi-trait-based estimation of growth rate and STAY. Therefore, the genetic correlations studied showed no antagonism of social effect with longevity. We recommend including social effects as selection criteria in breeding programs for achieving satisfactory genetic changes in both growth and longevity.

## Figures and Tables

**Table 1:** Distribution of observations among traits in both Landrace and Yorkshire pigs

Trait	ADG	STAY	NBA1
ADG	33,120	-	-
STAY	6,618	10,189	-
NBA1	7,579	10,189	11,654

ADG, average daily gain; STAY, stayability up to farrowing for a second litter; NBA1, number of piglets born alive in the first litter.

**Table 2:** Posterior means (posterior standard deviation) for each trait in Yorkshire pigs

Parameter	ADG	STAY	NBA1
$\sigma_{a_{D}}^{2}$	2,437 (151)	0.10 (0.03)	0.80 (0.12)
$\sigma_{a_{S}}^{2}$	18 (5)	-	-
$\sigma_{p}^{2}$	7,163 (88)	1.29 (0.06)	7.87 (0.11)
$\sigma_{TBV}^{2}$	3,464 (395)	-	-
*h*^2^	0.34 (0.02)	0.07 (0.02)	0.10 (0.01)
*T*^2^	0.48 (0.05)	-	-
$\sigma_{g}^{2}$	298 (45)	-	-
$\sigma_{l}^{2}$	249 (29)	0.19 (0.04)	0.17 (0.06)
$\sigma_{k}^{2}$	32 (5)	-	-
$\sigma_{e}^{2}$	3,816 (91)	1.00 (0.01)	6.90 (0.13)
*σ_a_*_*_DS_*_	5 (23)	-	-
*σ_lk_*	38 (9)	-	-

ADG, average daily gain; STAY, stayability up to farrowing for a second litter; NBA1, number of piglets born alive in the first litter; $\sigma_{a_{D}}^{2}$, direct genetic variance; $\sigma_{a_{S}}^{2}$, social genetic variance; $\sigma_{p}^{2}$, phenotypic variance; $\sigma_{TBV}^{2}$, total heritable variance; $h^{2}=\sigma_{a_{D}}^{2}/\sigma_{p}^{2}$, classical heritability; $T^{2}=\sigma_{TBV}^{2}/\sigma_{p}^{2}$, total heritability for model including social genetic effects; $\sigma_{g}^{2}$, random group variance; $\sigma_{l}^{2}$, random birth litter variance; $\sigma_{k}^{2}$, random social early-life environmental variance; $\sigma_{e}^{2}$, residual variance; *σ_a_*_*_DS_*_, covariance between direct and social genetic effects; *σ_lk_*, covariance between birth litter and social early-life environmental effects.

**Table 3:** Posterior estimates of genetic correlations among traits in Yorkshire pigs

Trait	Mean (SD)	95% HPD	Monte Carlo error
DBV_ADG_-SBV_ADG_	0.03 (0.11)	−0.18 to 0.24	0.010
DBV_ADG_-DBV_STAY_	−0.30 (0.13)	−0.55 to −0.04	0.011
DBV_ADG_-DBV_NBA1_	−0.04 (0.07)	−0.18 to 0.11	0.003
DBV_STAY_-DBV_NBA1_	0.31 (0.16)	0.01 to 0.61	0.017
SBV_ADG_-DBV_STAY_	0.04 (0.32)	−0.52 to 0.64	0.092
SBV_ADG_-DBV_NBA1_	0.27 (0.17)	−0.05 to 0.60	0.021
TBV_ADG_-DBV_STAY_	−0.23 (0.20)	−0.60 to 0.18	0.051
TBV_ADG_-DBV_NBA1_	0.11 (0.11)	−0.11 to 0.34	0.013

SD, standard deviation; 95% HPD, highest posterior density interval containing 95% of the observations; DBV_ADG_, direct breeding value for average daily gain; SBV_ADG_, social breeding value for average daily gain; DBV_STAY_, stayability up to farrowing for a second litter; DBV_NBA1_, number of piglets born alive in the first litter; TBV_ADG_, total breeding value for average daily gain.

**Table 4:** Posterior estimates of the litter environmental correlations between traits in Yorkshire pigs

Trait	Mean (SD)	95% HPD	Monte Carlo error
D_ADG_-S_ADG_	0.43 (0.11)	0.23 to 0.66	0.023
D_ADG_-D_STAY_	−0.38 (0.13)	−0.62 to −0.13	0.017
D_ADG_-D_NBA1_	0.07 (0.18)	−0.29 to 0.42	0.017
D_STAY_-D_NBA1_	−0.05 (0.22)	−0.47 to 0.38	0.028
S_ADG_-D_STAY_	0.09 (0.16)	−0.20 to 0.42	0.038
S_ADG_-D_NBA1_	0.05 (0.27)	−0.49 to 0.55	0.055

SD, standard deviation; 95% HPD, highest posterior density interval containing 95% of the observations; D_ADG_, direct litter effect for average daily gain; S_ADG_, social early-life effect for average daily gain; D_STAY_, direct litter effect for stayability up to farrowing for a second litter; D_NBA1_, direct litter effect for number of piglets born alive in the first litter.
